# A Depleting Anti-CD45 Monoclonal Antibody as Isolated Conditioning for Bone Marrow Transplantation in the Rat

**DOI:** 10.1371/journal.pone.0154682

**Published:** 2016-05-03

**Authors:** Mark D. Jäger, Florian W. R. Vondran, Wolf Ramackers, Tilmann Röseler, Hans J. Schlitt, Hüseyin Bektas, Jürgen Klempnauer, Kai Timrott

**Affiliations:** 1 Klinik für Allgemein-, Viszeral- und Transplantationschirurgie, Medizinische Hochschule Hannover, Hannover, Germany; 2 Klinik und Poliklinik für Chirurgie, Klinikum der Universität Regensburg, Regensburg, Germany; Emory University School of Medicine, UNITED STATES

## Abstract

**Objective:**

A monoclonal antibody (mAb) against the leukocyte common antigen CD45 (RT7 in rats) could facilitate bone marrow transplantation (BMT). This study in rats evaluates a depletive rat anti-RT7^a^ mAb as isolated tool for BMT conditioning without using irradiation or any chemotherapeutic / immunosuppressive agent.

**Methods:**

The model used a CD45 di-allelic polymorphism (RT7^a^/RT7^b^). The anti-RT7^a^ mAb was intravenously administered to LEW.1W rats (RT1^u^RT7^a^) at 5, 10 and 15 mg/kg. 1x10^8^ BM cells of MHC syngeneic (RT1^u^), MHC disparate (RT1^l^) or MHC haploidentical (RT1^u/l^) donors were transplanted. All BM donor strains carried the RT7^b^ allele so that their CD45^+^ cells were not affected by the anti-RT7^a^ mAb. Recipients were monitored for reconstitution and donor-chimerism in blood leukocytes.

**Results:**

mAb dosages of 5 or 10 mg/kg were myelosuppressive, whereas 15 mg/kg was myeloablative. Multi-lineage donor-chimerism at day 100 indicated engraftment of MHC syngeneic BM after any used mAb dosage (5 mg/kg: 46+/-7%; 10 mg/kg: 62+/-5%; 15 mg/kg: 80+/-4%). MHC disparate BM resulted in autologous reconstitution after conditioning by 10 mg/kg of the mAb and caused transient chimerism ending up in death associated with aplasia after conditioning by 15 mg/kg of the mAb. MHC haploidentical BM (F1 to parental) engrafted only after conditioning by 15 mg/kg (chimerism at day 100: 78+/-7%). Abandonment of α/β TCR^+^ cell depletion from BM grafts impaired the engraftment process after conditioning using 15 mg/kg of the mAb in the MHC syngeneic setting (2 of 6 recipients failed to engraft) and the MHC haploidentical setting (3 of 6 recipients failed).

**Conclusion:**

This depletive anti-RT7^a^ mAb is myelosuppressive and conditions for engraftment of MHC syngeneic BM. The mAb also facilitates engraftment of MHC haploidentical BM, if a myeloablative dose is used. RT7^b^ expressing, BM-seeded α/β TCR^+^ cells seem to impair the engraftment process after myeloablative mAb conditioning.

## Introduction

The leukocyte common antigen CD45 (RT7 in the rat) is widely expressed in the hematopoietic system. All mature leukocytes including tissue-seeded lymphocytes and many bone marrow (BM) seeded precursor cells express CD45 [1–3]. Even hematopoietic stem cells (HSC) show a weak CD45 expression and might be detected by anti-CD45 monoclonal antibodies (mAb) in different species [4–7].

Thus, an anti-CD45 mAb could be a beneficial tool for conditioning for bone marrow transplantation (BMT) [2]. So far, only few anti-CD45 mAb clones were tested for their potential to condition for BMT. In mice, a cytolytic rat anti-CD45 mAb (30F11) penetrated into BM and bound to BM seeded progenitor cells including CD34^+^ and Sca-1^+^ HSC [5]. This mAb was immunosuppressive and improved the engraftment of MHC disparate BM in recipients which were conditioned by a myelosuppressive dosage of total body irradiation (TBI) with 8 Gy. A myelosuppressive effect or even support for BMT conditioning was not reported for this mAb.

The complement-fixing rat anti-human CD45 mAb clones (YTH24.5 and YTH54.12) strongly reduced mature leukocytes and leukemic blasts in BM [8]. Analysis of BM aspirates as well as colony assays pre- and post-mAb-treatment did not reveal relevant effects on myeloid precursor cells. Nevertheless, these mAbs were successfully used in an antibody-based minimal-intensity conditioning regimen as myelosuppressive agents next to alemtuzumab (anti-CD52), fludarabine and low dose cyclophosphamide [9].

Anti-CD45 mAb were radiolabeled to target irradiation on BM seeded cells. A ^131^I-anti-CD45 conjugate delivered myelo- and immunosuppressive effects on BM level, so that MHC syngeneic BM could be successfully transplanted without any additional conditioning in mice [10]. In a H2-mismatched BMT setting it partially replaced TBI. Another radioconjugate (^213^Bi-anti-CD45) replaced 2 Gy TBI in a DLA-marrow transplantation model [11]. In humans, radiolabeled anti-CD45 antibodies were mainly used to reduce the leukemic burden in combination with non-myeloablative and reduced-intensity conditioning regimen [12].

We introduce here a rat anti-rat CD45 mAb (anti-RT7^a^ mAb), which strongly depletes T-lymphocytes, NK cells as well as granulocytes in blood and induces long-term acceptance of MHC disparate heart grafts in a rat model [13]. Furthermore, our group showed that this anti-RT7^a^ mAb can effectively deplete BM precursor cells of myeloid, T-lymphocyte, and thrombocytic lineage or even HSC when applied in high dosages to LEW.1W rats [6]. This mAb was also successfully used to eradicate hematopoietic chimerism in CD45 di-allelic rat models [6,14].

This study in rats uses also the CD45 di-allelic system in order to evaluate the rat anti-RT7^a^ mAb as tool for BMT conditioning in MHC syngeneic as well as MHC allogeneic settings. The major aim is to evaluate the potential of an anti-CD45 mAb as isolated tool for BMT conditioning without using irradiation or additional chemotherapeutic / immunosuppressive agents.

## Methods

### Animals

Used rat strains are given in Table 1. LEW.7B and LEW.1U-7B rats were provided by K. Wonigeit (Hannover, Germany). LEW.1W rats and F1 generation from LEW.1U-7B x LEW.7B were bred at the Zentrales Tierlaboratorium, Medizinische Hochschule Hannover. All animal procedures were approved by the Niedersächsisches Landesamt für Verbraucherschutz und Lebensmittelsicherheit (animal protection number 02/528). Animals were kept in a specific pathogen free facility in a circadian rhythm of light and dark cycle with free access to water and food. Ketamine and xylocain adopted to the actual body weight of every animal were applied for anaesthetics. Animals were visited daily. The animals were sacrificed by carbon dioxide inhalation and subsequent neck fracture at the endpoint of the follow-up. Respectively, this euthanizing procedure was used for all animals that acquired a poor clinical state (amongst others defined by loss of activity, increased sleepiness, reduced food intake) or to the defined end points of the experiments.

**Table 1:** MHC (RT1) and CD45 (RT7) immunogenetics of strain combinations used for the bone marrow transplantation model.

	Rat strain	MHC haplotype	CD45 allotype
		RT1	RT7
Recipient	LEW.1W	u	a
MHC syngeneic BM donor	LEW.1U-7B	u	b
MHC disparate BM donor	LEW.7B	l	b
MHC haploidentical BM donor	F1 (LEW.1U-7B x LEW.7B)	u / l	b

The recipient strain carries the RT7^a^ allele and is susceptible for the anti-RT7^a^ mAb treatment. All BM donor strains carry the RT7^b^ allele so that their CD45^+^ cells will not be affected by the anti-RT7^a^ mAb. MHC syngeneic BM donors carry the identical RT1 haplotype (RT1^u^) like LEW.1W recipients. MHC disparate BM donors were homogenously allogeneic in MHC (RT1^l^) alloantigens compared to the recipient strain. MHC haploidentical BM donors were chosen in the F1-to-parental direction, thus sharing the RT1^u^ haplotype of the recipient strain in one allele, but carrying the allogeneic RT1^l^ haplotype in the other allele.

### Monoclonal antibody against the RT7^a^ (CD45.1) antigen

The rat anti-RT7^a^ mAb is of IgG2_b_ isotype and detects the RT7.1 determinant of the RT7 antigen (CD45). This mAb was developed, produced and provided by K. Wonigeit (Hannover, Germany). Culture supernatant from the hybridoma producing anti-RT7^a^ mAb was purified by affinity chromatography using Protein-G 4 Fast Flow (Pharmacia, Schweden). Purified mAb was concentrated by Amicon Ultra centrifugation (Millipore Corporation, Ireland). The protein concentration of the pooled mAb was determined by Bradford method. The mAb was dissolved in phosphatase-buffered saline at a concentration of 2 mg/ml for intravenous injection. Former studies analysing the identical mAb clone in identical rat strain (LEW.1W) revealed that the mAb persists in serum for at least 14 days after intravenous injection [13]. The mAb was biotinylated for the biodistribution study.

### Procedures for studies of biodistribution and effects on the lympho-hematopoietic system

To enable reliable flowcytometric gating of lymphocyte subsets, which will be strongly depleted by the anti-RT7^a^ mAb, mixed chimeras carrying disparity only in the RT7 (CD45) antigen were generated (RT7^a^ / RT7^b^ chimerism). 1x 10^8^ bone marrow cells (BMC) from LEW.1W donors were transplanted into LEW.1U-7B recipients, which were conditioned by 7 Gy of TBI using a linear accelerator (Philips MU15F/225 kV, Hamburg, Germany). Thereby, a chimerism of LEW.1W-derived RT7^a^ leukocytes of about 30 percent were generated. At day 100 animals received biotinylated anti-RT7^a^ mAb (2 mg/kg). Analysis of thymus, lymph node, spleen, blood and BM were performed before and at days 3, 7, 14, 21 and 35 after mAb injection. Thereby, RT7^a^ positive fractions of lymphoid lineages (T-, B-lymphocytes and NK cells) as well as fractions of anti-RT7^a^ mAb coated cells were detected by flow cytometry.

The effect of the anti-RT7^a^ mAb on BM level was analysed in naive LEW.1W rats which received 10 mg/kg of the anti-RT7^a^ mAb intravenously. On day 7, animals were sacrificed, BM harvested from long bones and processed to flow cytometry and to histology.

### Bone marrow transplantation procedure

Male LEW.1W rats with a body weight between 180 and 220 gram received the anti-RT7^a^ mAb via the penile vein at dosages of 5, 10 or 15 mg/kg body weight.

Tissue analyses in former studies as well as in this study revealed that the antibody causes strong depletion of T-lymphocytes and NK cells as well as extensive coating of B-lymphocytes in lympho-hematopoietic compartments at day 3, so that the BMT was performed 3 days after application of the anti-RT7^a^ mAb [6,15]. Table 1 gives the strain combinations and MHC (RT1) haplotypes as well as CD45 (RT7) allotypes of MHC syngeneic, disparate and haploidentical BMT settings. BMC were harvested from long bones of euthanized donor rats. *In vitro* depletion of mature α/β TCR^+^ lymphocytes from BMC was performed using mouse anti-α/β TCR^+^ mAb (R73, mouse IgG_1_, generously provided by K. Wonigeit, Hannover, Germany) and immunomagnetic beads (Dynabeads M-450, goat anti-mouse IgG, Dynal Biotech GmbH, Hamburg, Germany) unless otherwise stated. BMC were suspended in PBS plus penicillin / streptomycin at a concentration of 1x 10^8^ BMC per ml.

BMC were injected into the penile vein at a dosage of 1 x 10^8^ vital, nucleated cells per recipient. Clinical signs of anemia like paleness of ears and eyes as well as symptoms of graft-versus-host disease including dermatitis, hair loss, loss of body weight, conjunctivitis, nasal bleeding, abdominal fullness and diarrhea were monitored three times a week. Weight indices were calculated by dividing the actual body weight through the one at baseline when the anti-RT7^a^ mAb was injected. Peripheral blood was weekly taken from the retroorbital plexus and was analysed using Sysmex XE2100 hematology analyzer (Diamond Diagnostics, USA). Donor chimerism within leukocytes of peripheral blood was determined using flow cytometry.

### Flow cytometry and bone marrow histology

Two- and 3-color antibody staining was carried out by standard protocols. Single cell suspensions of all examined organs were produced by cutting the parenchyma into small pieces and pushing it through nylon meshes. In addition erythrocytes were lysed by NH_4_Cl. Immunostaining was performed at 4°C on ice with optimal staining concentrations of following mAbs: anti-CD45RA (OX33, Becton Dickinson GmbH, Heidelberg, Germany), anti-granulocytes (HIS48, Becton Dickinson GmbH, Heidelberg, Germany), anti-CD45 (OX1, Becton Dickinson GmbH, Heidelberg, Germany), anti-CD45.2-Fitc, which detects the RT7^b^ allotype (HIS41, Becton Dickinson GmbH, Heidelberg, Germany), and anti-RT7^a^-biotin (RT7^a^, own production). The following antibodies were produced from the respective hybridomas (courtesy of Hüning, Würzburg, Germany; Williams, Oxford, United kingdom and Butcher, Cambridge, United Kingdom): anti-α/βTCR (R73), anti-NKR-P1 (3.2.3.). Following secondary mAbs respectively fluorochromes were used: goat-anti-mouse-PE, goat-anti-mouse-Fitc and SA-PE (all Becton Dickinson GmbH, Heidelberg, Germany). Blocking agents were normal rat serum as supplement to goat-anti-mouse mAbs and normal mouse serum.

All samples were analyzed on a FACS Calibur flow cytometer (Becton Dickinson Mountain View, USA).

Calculation of absolute lineage subset numbers in blood were based on the percentage of cells in the open wide gate, which represents total leukocyte number. The total leukocyte number per μl blood was kindly provided by the Abteilung für Hämatologie und Onkologie, Medizinische Hochschule Hannover.

For changes on BM level the density of CD45 expression and the extent of cellular granularity (SSC) were used to distinguish lymphoid and myeloid lineages. For histological analysis of BM smear compounds Giemsa-staining was used.

### Statistics

Statistical analysis was performed using IBM SPSS Statistics 21.0 (Chicago, USA) applying the unpaired *t* test for the comparison of characteristics. The data in the text and tables are presented as mean value ± standard deviation. The statistical significance was appointed at p<0.05.

## Results

### Biodistribution and effects of anti-RT7^a^ mAb in the lympho-hematopoietic system

**Fig 1:**
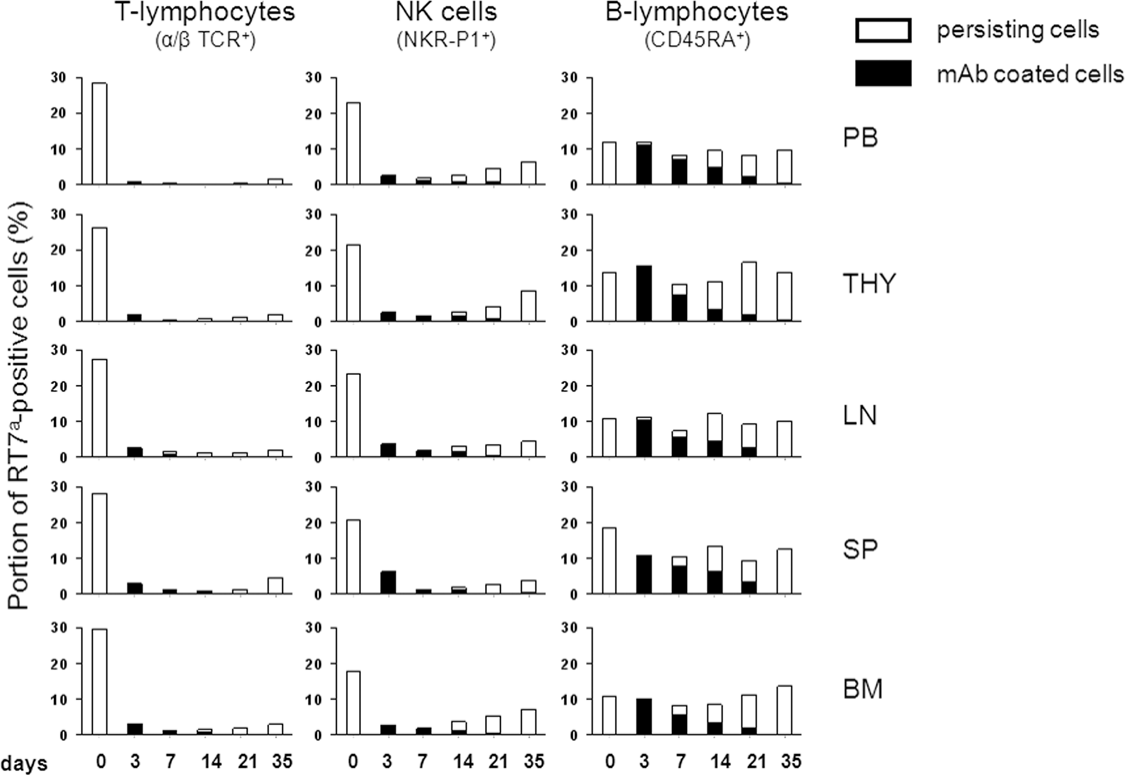
Biodistribution and effects of biotinylated anti-RT7^a^ mAb in lympho-hematopoietic compartments of RT7^a^/RT7^b^ chimeras. To enable reliable flowcytometric gating of lymphocyte subsets, which will be strongly depleted by the anti-RT7^a^ mAb, six mixed chimeras carrying disparity only in the RT7 (CD45) antigen were generated by sublethal TBI of 7 Gy. Reconstituted rats showed a multi-lineage RT7^a^-chimerism of 10 to 30 percent differing between lineages and compartments (day 0 = before mAb application). The biotinylated anti-RT7^a^ mAb was applied once (2 mg/kg) before the tissue compartments were analysed by flow cytometry at days 3, 7, 14, 21 and 35. The fraction of persisting RT7^a^ positive cells is given as percentage of all CD45^+^ cells per lineage (column height). The black column gives the fraction of RT7^a^ positive cells that is coated by the biotinylated anti-RT7^a^ mAb. T-lymphocytes (α/βTCR^+^) and NK cells (NKR-P1^+^) were strongly and compartment independently depleted, whereas B-lymphocytes (CD45RA^+^) were coated by anti-RT7^a^ mAb, but were not significantly reduced in cell number despite high-graded coating. (PB–peripheral blood, THY–thymus, LN–lymph node, SP–spleen, BM–bone marrow).

The species-identical rat anti-rat RT7^a^ mAb was biotinylated to monitor reliably the biodistribution by flow cytometry using streptavidin-conjugated fluorochrome. A prompt coating of mature T- and B-lymphocytes as well as NK cells was detected in all analysed compartments indicating an unexceptional distribution of the mAb into central and peripheral lympho-hematopoietic tissues (Fig 1). The coating persisted up to 21 days, whereby the percentage of coated cells decreased markedly after one week.

Chimeras carrying disparity in the RT7 (CD45) antigen were used to monitor the effects even in maturation compartments like thymus or BM. Sequential analysis revealed a prompt, strong and for up to 1 month persisting depletion of T-lymphocytes and NK cells in all analysed tissues (Fig 1). In contrast, percentages of B-lymphocytes were nearly unchanged despite high mAb coating rates.

**Fig 2:**
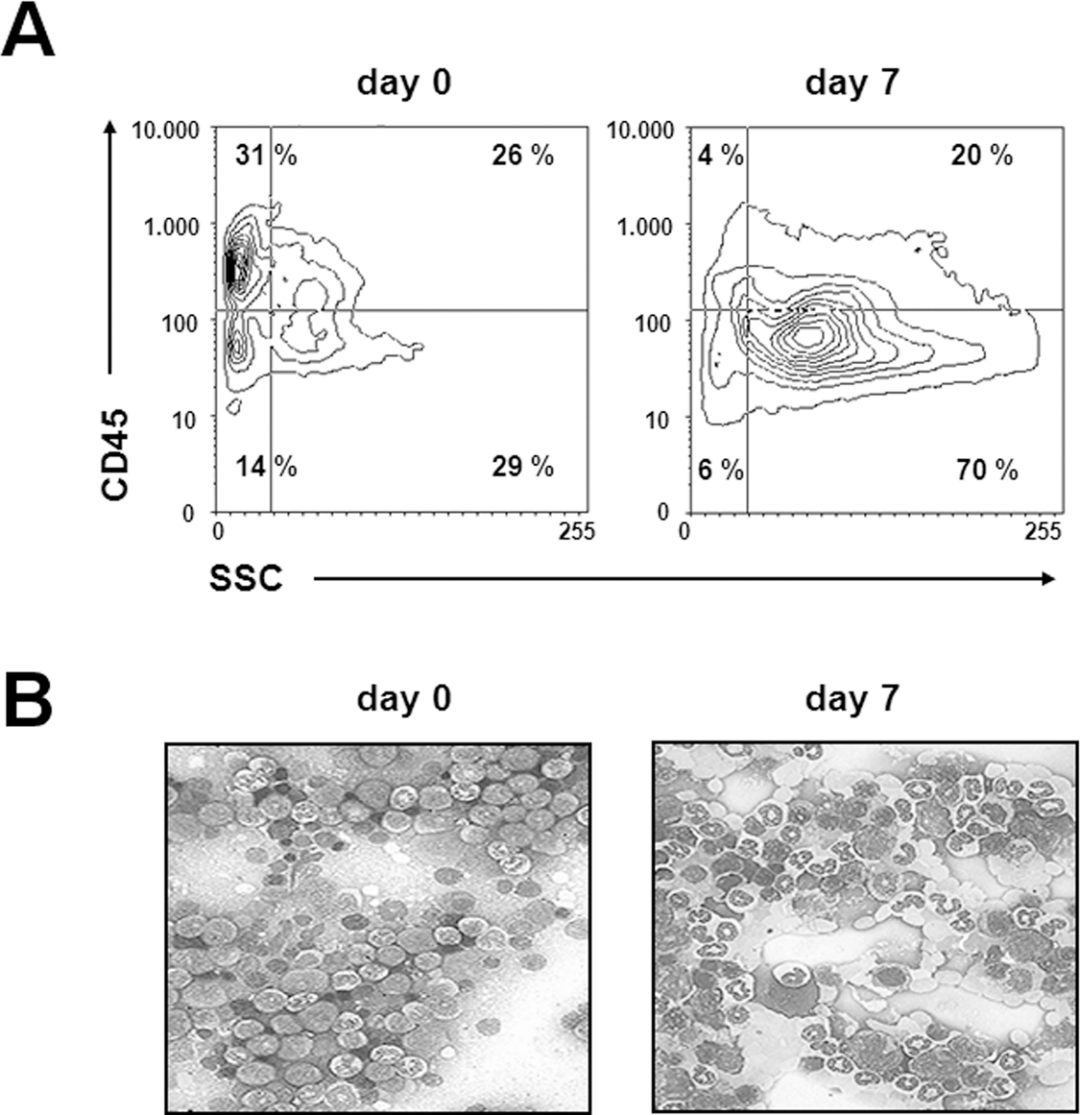
Effects of anti-RT7^a^ mAb on bone marrow level. A single dosage of 10 mg/kg of anti-RT7^a^ was injected into LEW.1W rats (n = 4). (A) BMC were analysed 7 days after mAb injection, whereby BMC were categorized by CD45 expression density and granularity using flow cytometry. Myelopoiesis showed a right shift, whereby SSC^low^/CD45^low^ early progenitors as well as SSC^low^/CD45^high^ lymphoid progenitors were markedly reduced. (B) Histology verified the right shift in myelopoiesis.

The effect on BM was analysed in normal LEW.1W rats receiving 10 mg/kg of the anti-RT7^a^ mAb. At day 7 a massive right shift in myelopoiesis (CD45^dim^ cells) and a relative reduction of CD45^high^ progenitors representing mainly lymphatic lineages occurred (Fig 2). The total count of harvested BMC per rat (6 long bones) was significantly reduced compared to control animals (day 7: 176 x 10^6^ ± 15 x 10^6^ vs. day 0: 308 x 10^6^ ± 25 x 10^6^; p < 0.01, n = 4 per group).

### Dosage dependent effects on hematopoiesis and outcome of MHC syngeneic BMT

**Fig 3:**
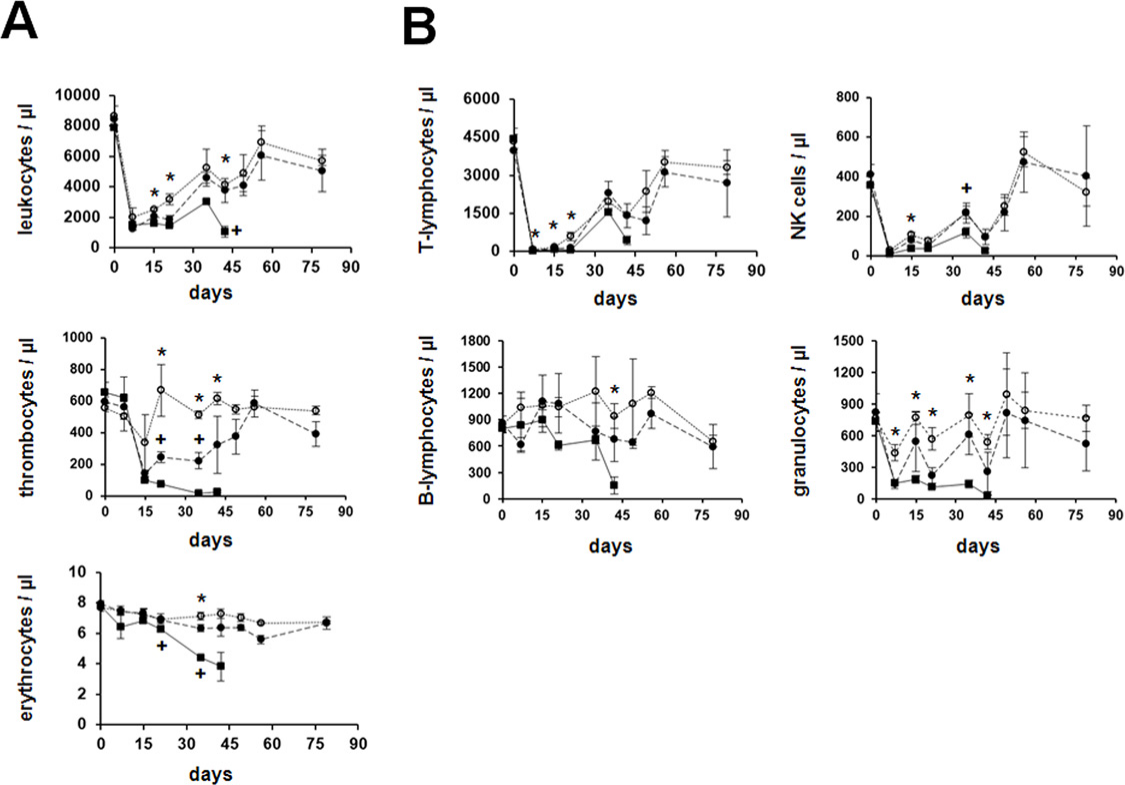
Dosage dependent effect of anti-RT7^a^ mAb on hematopoiesis and leukocyte lineages in blood. Anti-RT7^a^ mAb was intravenously injected at dosages of 5, 10 or 15 mg/kg into LEW.1W rats (RT7^a^, n = 4 per group). (A) Hematological parameters were regularly monitored and depicted as mean values +/- standard deviation per group (^…^○^…^ 5 mg/kg, —●—10 mg/kg,——■–—15 mg/kg). Animals, which received 15 mg/kg, died between days 36 and 43, whereas all other animals survived indefinitely. (B) Leukocyte lineages were analyzed by flow cytometry. Cell numbers of each subset were calculated and depicted as mean values +/- standard deviation per group (^…^○^…^ 5 mg/kg, —●— 10 mg/kg,——■–—15 mg/kg). Statistical analyses were performed applying the unpaired t test (* p < 0.05 (5 vs. 15 mg/kg), ^+^ p < 0.05 (10 vs. 15 mg/kg)).

The anti-RT7^a^ mAb was intravenously administered three days before BMT. Monitoring of the control groups, which did not receive BMT, showed that all dosages (5, 10 or 15 mg/kg) of the mAb caused a distinct reduction in leukocyte numbers after 1 week as well as in thrombocyte numbers after 2 weeks (Fig 3A). Cell number reductions were dependent from mAb dosages especially within thrombocytes. A dosage of 15 mg/kg caused severe pancytopenia resulting in fatal aplasia after five to six weeks so that all 4 control rats died (Fig 3A and Table 2). Another 5 control rats, which were treated with a mAb dosage of 15 mg/kg, received blood transfusions from identical rat strain (LEW.1W) at days 11, 15 and 18 (2ml unseparated blood at each date). These rescue transfusions could not prevent the death of all rats in association with clinical signs of aplasia at days 19, 26 (3x) and 28. A dosage of 10 mg/kg caused transient myelosuppression as indicated by thrombocytopenia between weeks 2 and 6 after application. Low dosage of 5 mg/kg caused still a considerable reduction in absolute leukocyte numbers, but no thrombocytopenia.

**Table 2:** Effect of anti-RT7^a^ mAb dosages on leukopoiesis and outcome of the MHC syngeneic BMT.

mAb	BMT	n	Survival	Leukocyte number (10^3^ cells /μl)			Donor chimerism (%)		
mg/kg	MHC-match		days	day 14	day 35	day 100	day 14	day 35	day 100
5	no BMT	4	> 200 x4	2.5 +/- 0.1	5.3 +/- 1.2	6.1 +/- 0.3	---	---	---
10	no BMT	4	> 200 x4	2.0 +/- 0.3	4.6 +/- 0.4	5.9 +/- 0.3	---	---	---
15	no BMT	4	36, 41 x2, 43	1.6 +/- 0.2	3.0 +/- 0.2		---	---	---
no mAb	syngeneic	6	> 200 x6	8.7 +/- 0.5	7.8 +/- 0.6	8.6 +/- 0.4	0.6 +/- 0.4	0	0
5	syngeneic	6	> 200 x6	3.6 +/- 0.3	8.5 +/- 1.2	7.7 +/- 1.2	14 +/- 9	22 +/- 6	46 +/- 7
10	syngeneic	6	> 200 x6	4.4 +/- 0.2	8.8 +/- 1.1	7.3 +/- 0.7	24 +/- 4	36 +/- 4	62 +/- 5
15	syngeneic	6	> 200 x6	6.1 +/- 1.2	6.5 +/- 1.9	6.9 +/- 0.5	43 +/- 10	61 +/- 7	80 +/- 4

LEW.1W (RT1^u^, RT7^a^) rats received anti-RT7^a^ mAb in different dosages as indicated. Control groups, who received no BMT, consisted of 4 animals each. BM grafts from LEW.1U-7B (RT1^u^, RT7^b^) donors underwent in vitro depletion of α/β TCR^+^ cells before 1x 10^8^ BMC were intravenously administered to LEW.1W recipients three days after mAb application (n = 6 per group). Animals were monitored for survival, leukocyte counts and donor-derived chimerism in CD45^+^ cells using anti-CD45 mAb (OX1) and donor-specific anti-RT7^b^ mAb (HIS41). Leukocyte numbers and percentages of donor chimerism are given as mean values +/- standard deviation at days 14, 35 and 100 after BMT.

Analysis of leukocyte lineages revealed that T-lymphocytes and NK cells were strongly and dosage dependently depleted (Fig 3B). Also granulocyte numbers were reduced dosage dependently, even though to a lower extent. In contrast, B-lymphocytes did not experience marked changes until pancytopenia occurred in animals treated with 15 mg/kg.

Stable chimerism developed in all groups of MHC syngeneic BMT. The degree of donor-derived leukocytes at day 100 after BMT increased parallel to the applied mAb dosages (Table 2). Controls receiving only the MHC syngeneic BM grafts, but no mAb conditioning, did not develop permanent chimerism.

### MHC mismatched BMT conditioned by anti-RT7^a^ mAb

MHC mismatched BMC did not engraft after conditioning with a non-myeloablative mAb dosage of 10 mg/kg as indicated by loss of the low grade donor chimerism in CD45^+^ cells in blood within 50 days (Table 3). All of these recipients reconstituted with recipient-derived leukopoiesis and survived at least 200 days. BMT outcome and overall survival was not different for recipients of MHC disparate or MHC haploidentical bone marrow grafts.

**Table 3:** MHC allogeneic BMT after conditioning by anti-RT7^a^ mAb.

mAb	BMT	n	Survival	Leukocyte number (10^3^ cells /μl)			Donor chimerism (%)		
mg/kg	MHC-match		days	day 14	day 35	day 100	day 14	day 35	day 100
10	disparate	6	> 200 x6	3.1 +/- 0.2	5.7 +/- 0.6	6.9 +/- 0.4	4 +/- 2	0.4 +/- 0.4	0
10	haploidentical	6	> 200 x6	3.6 +/- 0.5	6.2 +/- 0.6	7.6 +/- 1.2	6 +/- 3	1.1 +/- 0.5	0
15	disparate	11	26, 30 x2, 32 x4, 34 x2, 39, 41	4.7 +/- 0.8	2.3		36 +/- 16	33	
15	haploidentical	10	> 200 x10	13.8 +/- 0.9	7.1 +/- 0.3	8.3 +/- 0.2	74 +/- 11	55 +/- 12	78 +/- 7

LEW.1W recipients (RT1^u^, RT7^a^) received the anti-RT7^a^ mAb in a dosage of 10 mg/kg (non-myeloablative dosage) or 15 mg/kg (myeloablative dosage) 3 days prior to BMT. Numbers of animals vary between groups and are indicated (n). BM grafts of MHC disparate (LEW.7B: RT1^l^, RT7^b^) or MHC haploidentical (LEW.1U-7B x LEW.7B: RT1^u/l^, RT7^b^) donors were depleted from α/β TCR^+^ cells in vitro. 1x 10^8^ BMC were intravenously injected per recipient. Animals were monitored for survival, leukocyte counts and donor-derived chimerism in CD45^+^ cells using anti-CD45 mAb (OX1) and donor-specific anti-RT7^b^ mAb (HIS41). Leukocyte numbers and percentages of donor chimerism are given as mean values +/- standard deviation at days 14, 35 and 100 after BMT.

**Fig 4:**
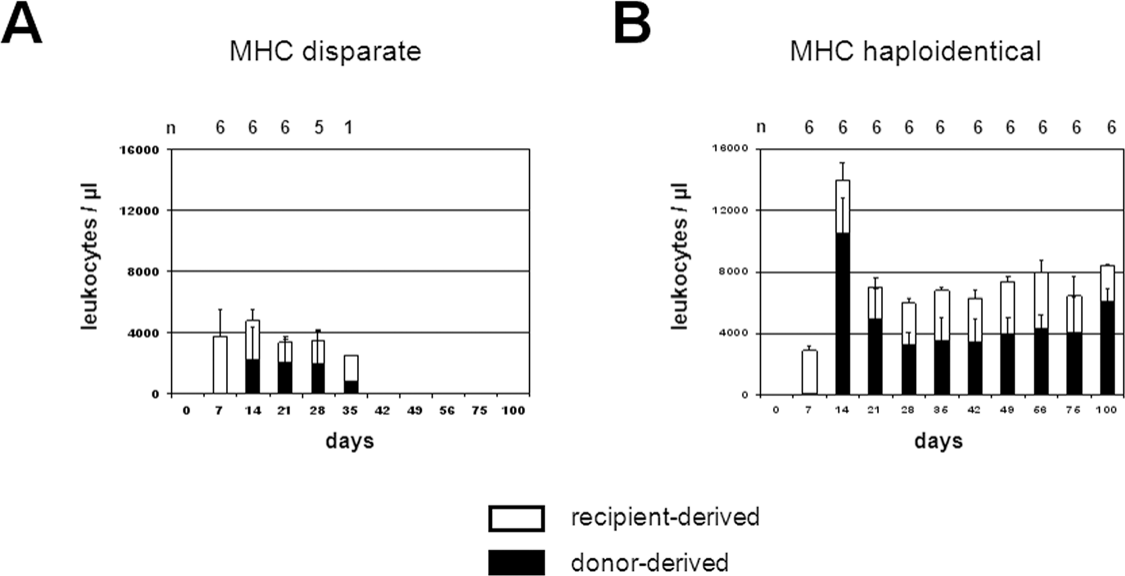
Reconstitution of donor and recipient derived leukocytes after MHC-disparate and MHC-haploidentical BMT conditioned by 15 mg/kg of the anti-RT7^a^ mAb. LEW.1W recipients (RT1^u^, RT7^a^, n = 6 per group) received anti-RT7^a^ mAb (15 mg/kg) 3 days prior to BMT. BMC of MHC disparate LEW.7B (RT1^l^, RT7^b^) and MHC haploidentical (LEW.1U-7B x LEW.7B (RT1^u/l^, RT7^b^)) donors were depleted from α/β TCR^+^ cells in vitro and intravenously injected (1x 10^8^ BMC per BMT). Numbers of surviving animals per group are indicated above the diagram. Total counts of leukocytes in peripheral blood are given. Donor- and host-derived fractions were determined by flow cytometry using a donor-specific anti-RT7^b^ mAb. Mean values of leukocytes from recipient origin (□) and from donor origin (■) are depicted. (A) Recipients of MHC disparate BMC lost chimerism over time, developed pancytopenia and died between days 26 and 41. (B) Recipients of MHC haploidentical BMC developed permanent chimerism and survived indefinitely.

When a myeloablative mAb dosage of 15 mg/kg was used recipients of MHC disparate as well as of MHC haploidentical BM grafts showed a considerable chimerism of donor-derived leukocytes at days 14, 21 and 28 (Fig 4). But long-term chimerism and permanent survival was only observed in recipients of MHC haploidentical BM whereas recipients of MHC disparate BM grafts lost donor chimerism, developed pancytopenia and died between days 26 and 41 (Table 3).

**Fig 5:**
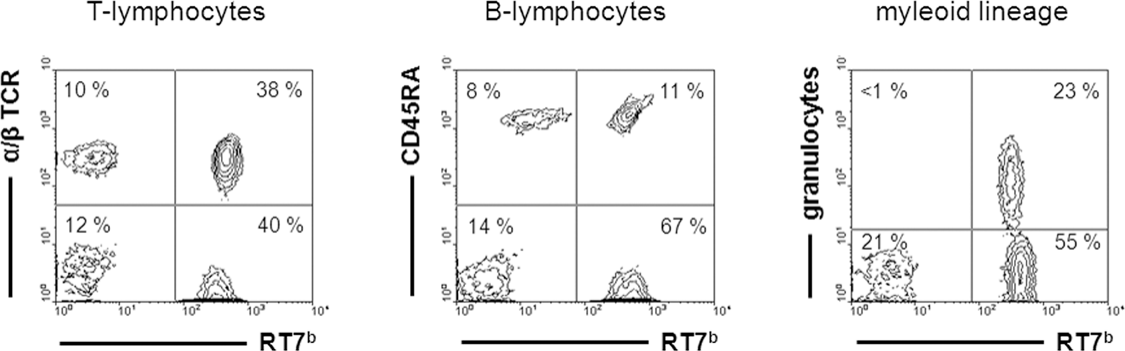
Multi-lineage chimerism in stable recipients of MHC haploidentical BM grafts conditioned by 15 mg/kg of anti-RT7^a^ mAb at day 200. Blood samples of long-term surviving recipients of MHC haploidentical BMT (F1 (LEW.7B x LEW.1U-7B: RT1^l/u^ RT7^b^) → LEW.1W: RT1^u^, RT7^a^) were analysed by flow cytometry for donor-derived chimerism (RT7^b^) within leukocyte lineages at day 200. Used mAbs were anti-α/β TCR mAb (R73) for T-lymphocytes, anti-CD45RA (OX33) for B-lymphocytes, anti-granulocytes (HIS48) for granulocytes and anti-RT7^b^ (HIS41) for donor-derived cells.

Healthy recipients of MHC haploidentical BMT showed long-term mixed chimerism at day 200 in T-lymphocytes (77 ± 6%), B-lymphocytes (61 ± 8%) and granulocytes (96 ± 2%) (Fig 5). Recipients of MHC disparate BM transiently developed predominantly donor-derived chimerism; at day 28 in granulocytes (82 ± 27%) and B-lymphocytes (35 ± 17%), but only low-level chimerism in T-lymphocytes (1.6 ± 0.8%).

Three additional recipients of MHC disparate BMT, which were conditioned by a myeloablative mAb dosage of 15 mg/kg, received a secondary MHC disparate BMT at day 28 (1x 10^8^ BMC after *in vitro* depletion of α/β TCR^+^ cells). These animals survived long-term and had a high-graded, multi-lineage donor-chimerism at day 100 in T-lymphocytes (65 ± 17%), B-lymphocytes (83 ± 1%) and granulocytes (99 ± 1%).

### Transplantation of BM without depletion of donor-derived α/β TCR^+^ cells

The final experiment tested the influence of BM resided donor-derived α/β TCR^+^ cells on the BM engraftment process after mAb conditioning using 15 mg/kg. Therefore, BM grafts did not undergo *in vitro* depletion of α/β TCR^+^ cells. The amounts of α/β TCR^+^ cells per untreated 1x 10^8^ BMC differed naturally between used donor strains (MHC syngeneic: 5.4 x 10^6^; MHC haploidentical: 5.1 x 10^6^; MHC disparate: 4.2 x 10^6^ α/β TCR^+^ cells). Control groups received BM grafts, which were depleted of α/β TCR^+^ cells *in vitro* like in previous experiments (Table 4).

**Table 4:** Transplantation of BM with or without depletion of donor-derived α/β TCR^+^ cells.

mAb	n	BMT	TCD	Amount	Anemia	Survival	Donor chimerism (%)			Survival rate
				α/β TCR^+^	clinical sign					at day 100
mg/kg		MHC-match		# / 1x10^8^ BMC	day of incidence	days	day 14	day 28	day 100	
15	4	syngeneic	yes	1.6 x 10^5^		> 100 x4	77 +/- 6	66 +/- 9	90 +/- 2	4 / 4
15	6	syngeneic	no	54 x 10^5^	28, 35	37, 48, > 100 x4	62 +/- 17	52 +/- 12	75 +/- 14	4 / 6
15	4	haploidentical	yes	1.5 x 10^5^	47 x2, 53	> 100 x4	76 +/- 6	54 +/- 8	70 +/- 4	4 / 4
15	6	haploidentical	no	51 x 10^5^	28 x2, 35	38, 43, 53, > 100 x3	73 +/- 11	49 +/- 20	72 +/- 2	3 / 6
15	5	disparate	yes	1.6 x 10^5^	21, 25 x2, 28 x2	29, 30, 38 x2, 42	39 +/- 17	32 +/- 30		0 / 5
15	5	disparate	no	42 x 10^5^	21 x2, 25, 28 x2	30, 32, 35 x2, 37	7 +/- 7	17 +/- 4		0 / 5

LEW.1W recipients (RT1^u^, RT7^a^) received the anti-RT7^a^ mAb in a dosage of 15 mg/kg (myeloablative dosage) 3 days prior to BMT. Numbers of animals vary between groups and are indicated (n). BM grafts of MHC syngeneic (LEW.1U-7B: RT1^u^, RT7^b^), MHC haploidentical (LEW.1U-7B x LEW.7B: RT1^u/l^, RT7^b^) or MHC disparate (LEW.7B: RT1^l^, RT7^b^) donors were depleted from α/β TCR^+^ cells in vitro (TCD yes) or were used without in vitro depletion of α/β TCR^+^ cells (TCD no). The amount of α/β TCR^+^ cells per 1x 10^8^ BMC is indicated for each group. Recipients were monitored for paleness of ears and eyes as clinical signs of anemia three times a week. The day with first sign of anemia is indicated (day of incidence). Animals were monitored for survival and levels of donor-derived chimerism in CD45^+^ cells using anti-CD45 mAb (OX1) and donor-specific anti-RT7^b^ mAb (HIS41). Percentages of donor chimerism are given as mean values +/- standard deviation at days 14, 28 and 100 after BMT.

**Fig 6:**
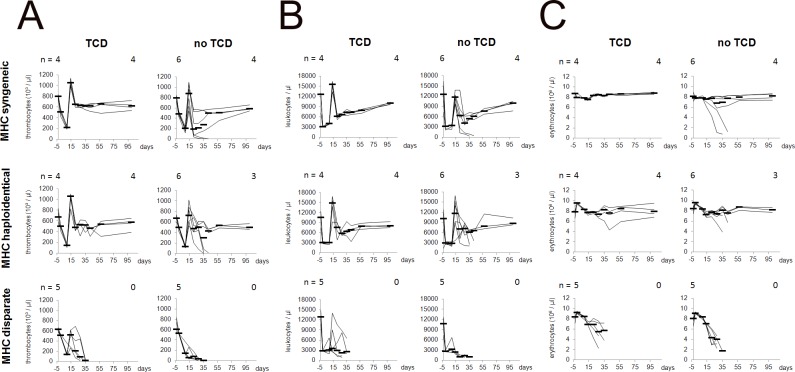
Hematological reconstitution of recipients receiving BM with or without depletion of donor-derived α/β TCR^+^ cells. LEW.1W (RT1^u^, RT7^a^) recipients were conditioned by 15 mg/kg of anti-RT7^a^ mAb 3 days prior to transplantation of 1 x 10^8^ BMC from MHC syngeneic (LEW.1U-7B: RT1^u^, RT7^b^), MHC haploidentical (LEW.1U-7B x LEW.7B: RT1^u/l^, RT7^b^) or MHC disparate (LEW.7B: RT1^l^, RT7^a^) donors. BM grafts were depleted from α/β TCR^+^ cells in vitro (TCD) or were kept untreated (no TCD). Thus, 1 x 10^8^ BMC contained 5.4 x 10^6^ α/β TCR^+^ cells (MHC syngeneic), 5.1 x 10^6^ α/β TCR^+^ (MHC haploidentical) respectively 4.2 x 10^6^ α/β TCR^+^ cells (MHC disparate) when untreated. Numbers of α/β TCR^+^ cells were 26 to 33 times lower after in vitro depletion 1.6 x 10^5^ α/β TCR^+^ cells in the MHC syngeneic and the MHC disparate setting and 1.5 x 10^5^ α/β TCR^+^ in the MHC haploidentical setting. (A) Courses of thrombocytes, (B) leukocytes and (C) erythrocytes are given with each animal represented by a single line. Numbers of animals entering the experiment (day -3) as well as those surviving at least 100 days are given above each diagram for the individual BMT setting. Animals, which did not survived 100 days after BMT, died at following days: MHC syngeneic BMT without TCD: days 37 and 48; MHC haploidentical BMT without TCD: days 38, 43 and 53; MHC disparate withTCD: days 29, 30, 38 (2x) and 42; MHC disparate without TCD: days 30, 32, 35 (2x) and 37. Mean of cell counts are given (–).

The larger amount of donor-derived α/β TCR^+^ cells appeared to impair the survival rates in MHC haploidentical as well as in MHC syngeneic BMT settings (Table 4). 2 of 6 recipients in the MHC syngeneic setting and 3 of 6 recipients in the MHC haploidentical setting, respectively, developed clinical signs of anemia just as all animals of the MHC disparate setting. These animals lost donor chimerism starting at day 28 and died with signs of aplasia (Table 4) as proven by hematological monitoring (Fig 6).

**Fig 7:**
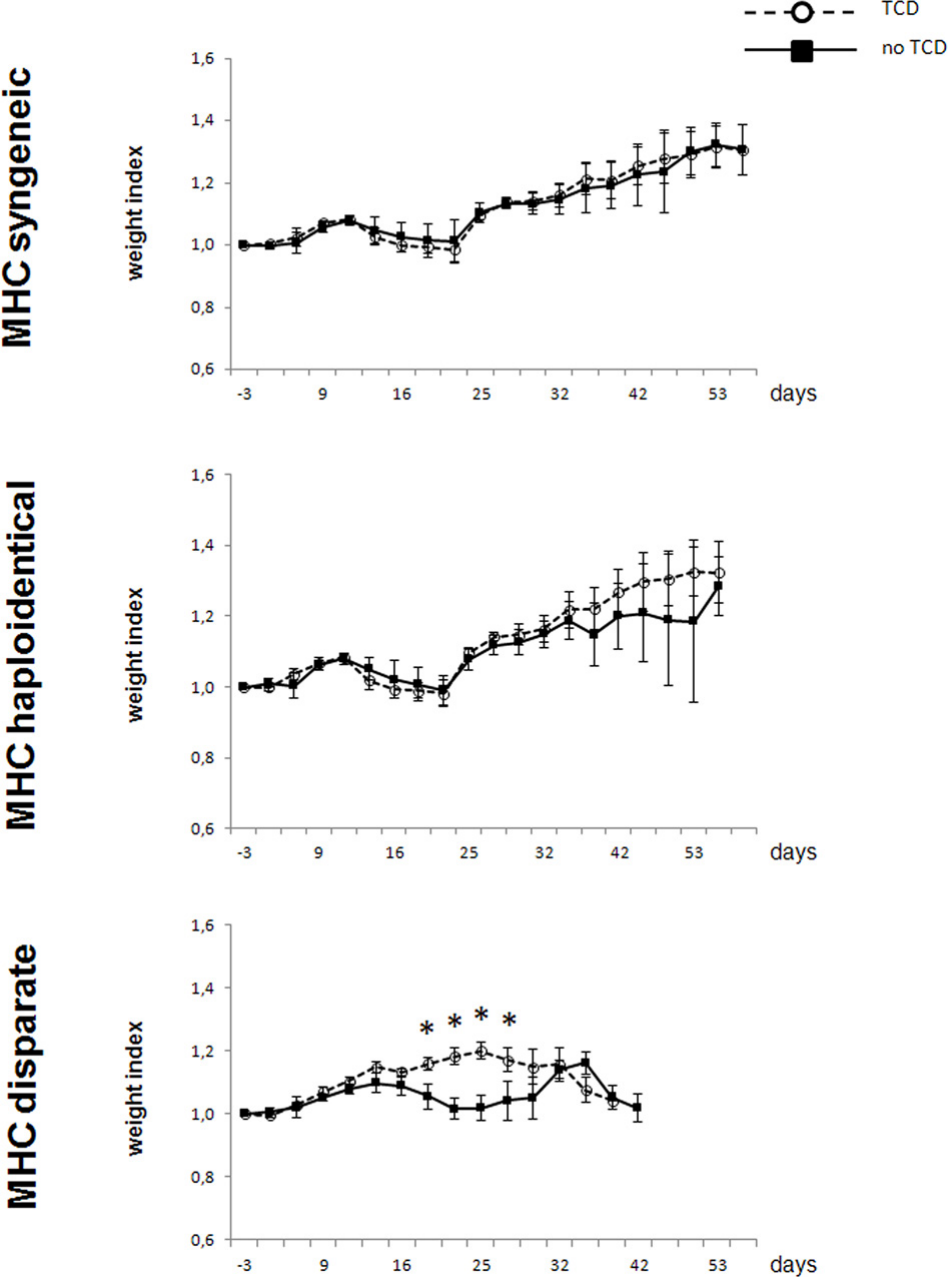
Weight indices of recipients receiving BM with or without depletion of donor-derived α/β TCR^+^ cells. *LEW*.*1W (RT1*^*u*^, *RT7*^*a*^*) recipients were conditioned by 15 mg/kg of anti-RT7*^*a*^ *mAb 3 days prior to transplantation of 1 x 10*^*8*^ *BMC from MHC syngeneic (LEW*.*1U-7B*: *RT1*^*u*^, *RT7*^*b*^*)*, *MHC haploidentical (LEW*.*1U-7B x LEW*.*7B*: *RT1*^*u/l*^, *RT7*^*b*^*) or MHC disparate (LEW*.*7B*: *RT1*^*l*^, *RT7*^*a*^*) donors*. *BM grafts were depleted from α/β TCR*^*+*^ *cells in vitro (TCD) or were kept untreated (no TCD)*. *Thus*, *1 x 10*^*8*^ *BMC contained 5*.*4 x 10*^*6*^ *α/β TCR*^*+*^ *cells (MHC syngeneic)*, *5*.*1 x 10*^*6*^ *α/β TCR*^*+*^ *(MHC haploidentical) and 4*.*2 x 10*^*6*^ *α/β TCR*^*+*^ *cells (MHC disparate)*, *respectively*, *when untreated*. *Weight indices were calculated by dividing the actual weight of the recipients through the baseline weight at the time point of BMT conditioning (= anti-RT7*^*a*^ *mAb injection)*. *Weight indices were calculated and depicted as mean values +/- standard deviation per group (*^*…*^*○*^*…*^ *depletion of* α/β TCR^+^ cells; –*■– no depletion of* α/β TCR^+^ cells) Statistical analyses were performed applying the unpaired t test (* p < 0.*05)*.

Clinical signs of GvHD were not observed in the MHC syngeneic and MHC haploidentical settings. Animals of the MHC disparate setting, which received BM inocula without *in vitro* depletion of α/β TCR^+^ cells, developed abdominal fullness and diarrhea. Consequently their weight indices were significantly lower compared to those of animals receiving MHC disparate BM after *in vitro* depletion of α/β TCR^+^ cells (Fig 7).

## Discussion

The applied rat anti-RT7^a^ mAb causes a dosage dependent myelosuppression in LEW.1W rats. A high mAb dosage of 15 mg/kg can even cause death of the rats in association with an aplasia-like syndrome. Repeated transfusions of whole blood from identical donors could not rescue the animals from death. Thus, a sustained damage of early bone marrow precursor cells or even HSC could be assumed. This assumption is in line with previous experiments of our group, that revealed the potential of the anti-RT7^a^ mAb to effectively deplete BM precursor cells of myeloid, T-lymphocyte, and thrombocytic lineage or even HSC using different experimental settings [6].

Other unconjugated lytic anti-CD45 mAbs, which were administered to mice (rat anti-mouse CD45; 30F11) or humans (rat anti-human CD45; YTH24.5 and YTH54.12), showed persistent effects on lymphopoiesis and transient effects on myelopoiesis, but no significant effect on marrow progenitors or HSC [5,6,8,16]. Interestingly, the lytic rat anti-mouse CD45 mAb (30F11) penetrated into the bone marrow and achieved binding levels that were proportional to the degree of CD45 antigen expression by BM progenitor cells including CD34^+^ and Sca-1^+^ HSC, but did not reduce the number and functionality of BM precursor cells when administered intraperitoneally with a total dosage of 4 mg/kg body weight [5]. Also, the cytolytic rat anti-human CD45 mAbs (YTH24.5 and YTH54.12), which bind to contiguous but non-overlapping epitopes of the CD45 molecule and work synergistically to recruit lytic cellular mechanisms, reduced lymphoid, myeloid and leukemic blasts, but largely spared BM progenitors when applied at a total dosage of 1.6 mg/kg body weight [8,16]. Since we used comparatively high mAb dosages in respect to the cited mAb clones analysed in mice and humans, we cannot exclude that the myelosuppression by the anti-RT7^a^ mAbcould be rather associated with mAb dosing than any differences in species-specific CD45 expression on BMC, differences in the epitopes detected by these anti-CD45 mAbs or in biodistribution as well as mechanisms of cell depletion caused by the different anti-CD45 mAb clones.

Nevertheless, our study showed that our lowest used mAb dosage (5 mg/kg) enabled for reliable engraftment of MHC syngeneic BM. Since identical BMC numbers failed to engraft in untreated recipients, we assume that this anti-RT7^a^ mAb already affects host HSC in a similar manner to that of whole body irradiation with 1 to 3 Gy in respect to achieving engraftment of donor HSC [17,18]. Thus, we showed that even low dosages of this depleting anti-RT7^a^ mAb enabled for competitive engraftment of donor-derived HSC next to host-derived ones [19]. Furthermore, we observed that the degree of the long-term, multi-lineage chimerism increased parallel to the administered mAb dosage in the MHC syngeneic BMT setting. Similar linearly increasing degrees of leukocyte chimerism were also reported in murine models that used increasing intensities of conditioning by chemotherapeutics, total body irradiation or radiolabeled anti-CD45 mAbs in MHC syngeneic settings [10,20–22]. Thus, we assume that the rat anti-RT7^a^ mAb conditions for engraftment of donor-derived HSC in a dosage dependent manner like other conditioning tools do.

So far, experiments testing the ability for isolated conditioning by an anti-CD45 mAb are published only for the rat anti-mouse CD45 mAb (30F11) [5,8]. There, the anti-CD45 mAb was intraperitoneally administered to C57Bl6J mice at a total dosage of 4 μg/g over 4 days. In contrast to our rat model, the MHC syngeneic BMT in mice was not successful, although a comparable amount of the anti-CD45 mAb (mice: 4 μg/g; rat: 5 mg/kg) and a relatively higher number of BMC per gram body weight were used in the murine model (up to 4x 10^7^ BMC per mouse; 1x 10^8^ BMC per rat). This lack of the anti-mouse CD45 mAb clone (30F11) to support engraftment of MHC syngeneic HSC is in line with reported facts that the 30F11 clone did not relevantly decrease number and functionality of BM precursor cells and did not improve the development of chimerism after conditioning by total body irradiation with 5.5 Gy[5]. Thus, the major difference of our anti-RT7^a^ mAb tested in rats and the 30F11 clone tested in mice is the ability to condition for engraftment of MHC syngeneic HSC, if no chemotherapy or irradiation is added. Our anti-RT7^a^ mAb has also strong immunosuppressive properties as shown in former experiments using the identical rat strain combination (LEWIS to LEW.1W: RT1^l^ to RT1^u^) where a single injection of 10 mg/kg body weight achieved long-term acceptance for MHC disparate heart grafts [13]. This strong immunosuppressive effect is based on an immediate and tremendous depletion of T-lymphocytes and NK cells in blood and lymphoid tissues [6,13].

Other anti-CD45 mAb clones, which were used in mice (30F11) or human beings (YTH24.5 and YTH54.12) likewise caused adistinct reduction of T-lymphocytes as well as NK cells and even of B-lymphocyteswhereas mature myeloid lineages in blood and lymphoid tissues were less affected [5,8,16]. These mAb clones provided functional immunossuppression in a MHC disparate BMT setting, but total body irradiation of 8 Gy or immunossupressive and chemotherapeutic drugs had to be added for successful BM engraftment.

Although our anti-RT7^a^ mAb did not deplete B-lymphocytes in contrast to the other anti-CD45 mAb clones, its isolated usage conditioned successfully for engraftment of MHC allogeneic BM. Thereby, we had to use higher mAb dosages compared to protocols inducing long-term acceptance of MHC disparate heart grafts using the identical rat strain combination. One reason for the requirement of such high anti-RT7^a^ mAb dosages could be that the long-term acceptance for heart grafts after anti-RT7^a^ mAb treatment is most likely maintained by an intragraft Th2-pattern [23]. In contrast durable experimental BM engraftment necessarily requires thymic engraftment ensuring central deletional tolerance [24]. In this light, we show here that our anti-RT7^a^ mAb penetrates into the thymus and depletes strongly RT7^a^ positive thymocytes. Furthermore, we observed that the detection of increasing numbers of donor-derived T-lymphocytes in blood, which might reflect thymic engraftment of donor cell lineages, was associated with successful BMT [25]. In this context, we showed that a mAb dosage of 15 mg/kg body weight was required for successful BMT in the MHC haploidentical setting. A dosage of 10 mg/kg body weight was indeed immunosuppressive as indicated by persistence of MHC mismatched leukocytes in peripheral blood over weeks, but it was not sufficient to guarantee durable BM engraftment and stable chimerism across a MHC allobarrier. Thus, we assume that successful MHC allogeneic BMT requires stronger immunosuppression than heart grafting across a MHC allobarrier when our depletive anti-RT7^a^ mAb is used as isolated conditioning tool. The strong immunosuppressive and myeloablative dosage of our anti-RT7^a^ mAb (15 mg/kg) guaranteed successful engraftment of MHC haploidentical BMC in the F1 to parental direction, but did not in the complete MHC disparate BMT setting. Although we cannot exclude any rejection of the allogeneic BM inocula by anti-donor allo-antibodies or persisting recipient-derived B-lymphocytes, this immunological mechanism seems unlikely since donor-derived leukocytes were detected in blood of the recipients for up to 35 days after allogeniec BMT (Table 3). Moreover, we used inbred rat strains without any allosensitization, so that we did not assume a primed humoral and cellular response against donor antigens [26]. In addition, the secondary “rescue” BMT, which was performed on day 28 using BMC of immunogenetic identically MHC disparate donors, resulted in stable chimerism. This fact shows that an allosensitization against MHC donor-antigens with impact to reject the secondary BM graft did not occur during the persistence of the anti-RT7^a^ mAb. It seems also unlikely that the process of HSC engraftment is impaired by residual NK cell mediated reactivity against MHC disparate BM, since a strong and tissue wide depletion of NK cells is caused by the anti-RT7^a^ mAb [27,28]. On the other hand, it could be possible that any graft-versus-host reactivity impairs the BM engraftment process in our model. To test this circumstance, we applied BM inocula with an increased amount of donor-derived α/β TCR^+^ cells and observed that the BM engraftment process was even impaired in some recipients of the MHC syngeneic but CD45 mismatched setting (2 of 6 animals lost chimerism and died). Also in the F1-to-parental MHC haploidentical setting, where any GvH-reactivity might only exist against the host-specific CD45 alloantigen (CD45.1) BM engraftment failed in 50 percent of recipients. Weight indices were not significantly reduced in both these settings as sign that the GvH-reactivity was not relevantly directed against epithelial tissues expressing MHC alloantigens. Thus, it seems possible that the combination of the strongly depletive anti-RT^a^ mAb and any GvH-reactivity even when restricted against CD45.1^+^ cells impairs the engraftment of CD45.2^+^ HSC.

Reflecting any mAb mediated disturbance of BM environment, we further observed that a delayed secondary BM graft of RT7^b^ positive and MHC disparate donors rescued anemic recipients and resulted in long-term survival with high-graded and stable donor-derived chimerism. This observation might underline that synchronization of strong cell depletion mediated by the anti-RT7^a^ mAb and of any alloreactivity directed against CD45 or even against MHC antigens could be inauspiciously for safe BM engraftment.

Taken together, our study represents a proof of principle that an anti-CD45 mAb could condition for successful BM engraftment even across a MHC allobarrier under artificial circumstances using a di-allelic CD45 antigen system. Our experimental system used a rat anti-RT7^a^ mAb which persists for up to 2 weeks in serum of the recipients. Thus, one has to keep in mind these specific features of our artificial system when transferring our observations in other systems. On the other hand, the di-allelic system offered the opportunity to analyse the coincidental effects of considerable depletion of CD45^+^ cells on bone marrow level and the infusion of allogeneic BMC including α/β TCR^+^ cells mediating probably any GvH reactivity.

In conclusion, we could show for the first time that isolated usage of a depletive rat anti-RT7^a^ mAb causes myelosuppression and conditions for successful HSC engraftment without addition of irradiation or any chemotherapeutic / immunosuppressive agent. The immunosuppressive properties of the anti-CD45 mAb enable also for BM engraftment across a full MHC allobarrier in a haploidentical setting (F1 to parental), but require mAb dosages with myeloablative potential. The application of such high mAb dosages could bring along the risk for disturbance of the BM microenvironment and HSC engraftment failure especially in interference with any GvH reactivity.

Thus in transfer, a strongly depletive and myelosuppressive anti-CD45 mAb could be a beneficial tool for eradication of host-derived CD45^+^ cells including BM seeded precursor cells or HSC, so that conditioning for HSC transplantation could be possible, if chemotherapy or irradiation should not be favoured e.g. in non-malignant disorders or tolerance induction by chimerism [29–32]. But any potential risk like severe damage on bone marrow level probably resulting from inconvenient interference of strong depletion of host CD45^+^ cells and GvH reactivity should be clarified before high dosages of depleting anti-CD45 mAb would be used in clinical settings.

## References

[1] Woodford-Thomas T, Thomas ML. The leukocyte common antigen, CD45 and other protein tyrosine phosphatases in hematopoietic cells. Semin Cell Biol 1993 12;4(6):409–418. 8305680 10.1006/scel.1993.1049

[2] Dahlke MH, Larsen SR, Rasko JE, Schlitt HJ. The biology of CD45 and its use as a therapeutic target. Leuk Lymphoma 2004 2;45(2):229–236. 15101706 10.1080/1042819031000151932

[3] Hermiston ML, Xu Z, Weiss A. CD45: a critical regulator of signaling thresholds in immune cells. Annu Rev Immunol 2003;21:107–137. 12414720 10.1146/annurev.immunol.21.120601.140946

[4] Gratama JW, Sutherland DR, Keeney M, Papa S. Flow cytometric enumeration and immunophenotyping of hematopoietic stem and progenitor cells. J Biol Regul Homeost Agents 2001 Jan-Mar;15(1):14–22. 11388740

[5] Wulf GG, Luo KL, Goodell MA, Brenner MK. Anti-CD45-mediated cytoreduction to facilitate allogeneic stem cell transplantation. Blood 2003 3 15;101(6):2434–2439. 12433683 10.1182/blood-2002-08-2379

[6] Dahlke MH, Lauth OS, Jager MD, Roeseler T, Timrott K, Jackobs S, et al In vivo depletion of hematopoietic stem cells in the rat by an anti-CD45 (RT7) antibody. Blood 2002 5 15;99(10):3566–3572. 11986209 10.1182/blood.v99.10.3566

[7] Wickenhauser C, Thiele J, Drebber U, Kvasnicka HM, Thiel A, Schmitz B, et al CD34+ human hemopoietic progenitor cells of the bone marrow differ from those of the peripheral blood: an immunocytochemical and morphometric study. Acta Haematol 1995;93(2–4):83–90. 7543721 10.1159/000204117

[8] Brenner MK, Wulf GG, Rill DR, Luo KL, Goodell MA, Mei Z, et al Complement-fixing CD45 monoclonal antibodies to facilitate stem cell transplantation in mouse and man. Ann N Y Acad Sci 2003 5;996:80–88. 12799286 10.1111/j.1749-6632.2003.tb03236.x

[9] Straathof KC, Rao K, Eyrich M, Hale G, Bird P, Berrie E, et al Haemopoietic stem-cell transplantation with antibody-based minimal-intensity conditioning: a phase 1/2 study. Lancet 2009 9 12;374(9693):912–920. 10.1016/S0140-6736(09)60945-4 19729196

[10] Matthews DC, Martin PJ, Nourigat C, Appelbaum FR, Fisher DR, Bernstein ID. Marrow ablative and immunosuppressive effects of 131I-anti-CD45 antibody in congenic and H2-mismatched murine transplant models. Blood 1999 1 15;93(2):737–745. 9885237

[11] Sandmaier BM, Bethge WA, Wilbur DS, Hamlin DK, Santos EB, Brechbiel MW, et al Bismuth 213-labeled anti-CD45 radioimmunoconjugate to condition dogs for nonmyeloablative allogeneic marrow grafts. Blood 2002 7 1;100(1):318–326. 12070043 10.1182/blood-2001-12-0322

[12] Storb R. Reduced-intensity conditioning transplantation in myeloid malignancies. Curr Opin Oncol 2009 6;21 Suppl 1:S3–5. 10.1097/01.cco.0000357467.45843.ba 19561410 PMC2895692

[13] Ko S, Jager MD, Tsui TY, Deiwick A, Dinkel A, Rohde F, et al Long-term allograft acceptance induced by single dose anti-leukocyte common antigen (RT7) antibody in the rat. Transplantation 2001 4 27;71(8):1124–1131. 11374414 10.1097/00007890-200104270-00020

[14] Ko S, Deiwick A, Jager MD, Dinkel A, Rohde F, Fischer R, et al The functional relevance of passenger leukocytes and microchimerism for heart allograft acceptance in the rat. Nat Med 1999 11;5(11):1292–1297. 10545996 10.1038/15248

[15] Ko S, Dahlke MH, Lauth O, Jager MD, Deiwick A, Dinkel A, et al Bone marrow aplasia induced by passenger leukocytes from heart allografts. Exp Hematol 2001 3;29(3):339–344. 11274762 10.1016/s0301-472x(00)00676-7

[16] Krance RA, Kuehnle I, Rill DR, Mei Z, Pinetta C, Evans W, et al Hematopoietic and immunomodulatory effects of lytic CD45 monoclonal antibodies in patients with hematologic malignancy. Biol Blood Marrow Transplant 2003 4;9(4):273–281. 12720220 10.1053/bbmt.2003.50024

[17] Koporc Z, Bigenzahn S, Blaha P, Fariborz E, Selzer E, Sykes M, et al Induction of mixed chimerism through transplantation of CD45-congenic mobilized peripheral blood stem cells after nonmyeloablative irradiation. Biol Blood Marrow Transplant 2006 3;12(3):284–292. 16503497 10.1016/j.bbmt.2005.11.011

[18] Tomita Y, Sachs DH, Sykes M. Myelosuppressive conditioning is required to achieve engraftment of pluripotent stem cells contained in moderate doses of syngeneic bone marrow. Blood 1994 2 15;83(4):939–948. 7906567

[19] Stewart FM, Zhong S, Wuu J, Hsieh C, Nilsson SK, Quesenberry PJ. Lymphohematopoietic engraftment in minimally myeloablated hosts. Blood 1998 5 15;91(10):3681–3687. 9573004

[20] Anam K, Black AT, Hale DA. Low dose busulfan facilitates chimerism and tolerance in a murine model. Transpl Immunol 2006 1;15(3):199–204. 16431286 10.1016/j.trim.2005.09.009

[21] Down JD, Tarbell NJ, Thames HD, Mauch PM. Syngeneic and allogeneic bone marrow engraftment after total body irradiation: dependence on dose, dose rate, and fractionation. Blood 1991 2 1;77(3):661–669. 1991176

[22] van Os R, Konings AW, Down JD. Radiation dose as a factor in host preparation for bone marrow transplantation across different genetic barriers. Int J Radiat Biol 1992 4;61(4):501–510. 1349332 10.1080/09553009214551261

[23] Jager MD, Tsui T, Aselmann H, Dahlke MH, Deiwick A, Neipp M, et al Features of tolerance achieved by antigen and a single injection of an anti-CD45 monoclonal antibody in rats. Transplant Proc 2001 Feb-Mar;33(1–2):142 11266748 10.1016/s0041-1345(00)01944-8

[24] Sykes M, Szot GL, Swenson KA, Pearson DA. Induction of high levels of allogeneic hematopoietic reconstitution and donor-specific tolerance without myelosuppressive conditioning. Nat Med 1997 7;3(7):783–787. 9212108 10.1038/nm0797-783

[25] Sykes M, Szot GL, Swenson K, Pearson DA, Wekerle T. Separate regulation of peripheral hematopoietic and thymic engraftment. Exp Hematol 1998 6;26(6):457–465. 9620278

[26] Taylor PA, Ehrhardt MJ, Roforth MM, Swedin JM, Panoskaltsis-Mortari A, Serody JS, et al Preformed antibody, not primed T cells, is the initial and major barrier to bone marrow engraftment in allosensitized recipients. Blood 2007 2 1;109(3):1307–1315. 17018854 10.1182/blood-2006-05-022772 PMC1785137

[27] Neipp M, Gammie JS, Exner BG, Li S, Chambers WH, Pham SM, et al A partial conditioning approach to achieve mixed chimerism in the rat: depletion of host natural killer cells significantly reduces the amount of total body irradiation required for engraftment. Transplantation 1999 8 15;68(3):369–378. 10459540 10.1097/00007890-199908150-00008

[28] Barao I, Murphy WJ. The immunobiology of natural killer cells and bone marrow allograft rejection. Biol Blood Marrow Transplant 2003 12;9(12):727–741. 14677112 10.1016/j.bbmt.2003.09.002

[29] Andreani M, Testi M, Lucarelli G. Mixed chimerism in haemoglobinopathies: from risk of graft rejection to immune tolerance. Tissue Antigens 2014 3;83(3):137–146. 10.1111/tan.12313 24571472

[30] Felfly H, Haddad GG. Hematopoietic stem cells: potential new applications for translational medicine. J Stem Cells 2014;9(3):163–197. doi: jsc.2014.9.3.163 25157450

[31] Kawai T, Sachs DH. Tolerance induction: hematopoietic chimerism. Curr Opin Organ Transplant 2013 8;18(4):402–407. 10.1097/MOT.0b013e328363621d 23838644

[32] Tolar J, Mehta PA, Walters MC. Hematopoietic cell transplantation for nonmalignant disorders. Biol Blood Marrow Transplant 2012 1;18(1 Suppl):S166–71. 10.1016/j.bbmt.2011.10.023 22226101

